# Supplementary material for: Protozoan and Rickettsial Pathogens in Ticks Collected from Infested Cattle from Turkey
Source: Pathogens. 2022 Apr 22;11(5):500. doi: 10.3390/pathogens11050500 (PMC9146054; doi:10.3390/pathogens11050500)
Supplement: Supplementary file 1 [file pathogens-11-00500-s001.zip › pathogens-1662639-supplementary.pdf]

Table S1. Tick species identification based on the mitochondrial *16S rDNA* gene.

Tick *16S rDNA* Sequence				Blastn Match	
**Accession Number**	**Length (bp)**	**Query Cover (%)**	**Identity (%)**	**Accession Number**	**Tick Species**
OL347853	452	100	99.78	KT391060	*Hyalomma marginatum*
OL347856	450	100	99.33	KR870972	*Hyalomma excavatum*
OL347855	448	100	100	MK829042	*Hyalomma anatolicum*
OL347857	453	89	99.50	MT229195	*Rhipicephalus turanicus*
OL347854	450	100	99.78	MT302761	*Rhipicephalus bursa*

Table S2. Primer sets used for the detection of tick-borne pathogens.

Pathogen Target gene	Type of PCR	Oligonucleotide Sequences (5'→ 3')	Amplicon Size	Reference
Tick 16S *rDNA*	Conventional	F - GCTCAATGATTTTTTAAATTGCTGT<br>R - CCGGTCTGAACTCAGATCAAGT	460	[44]
Theileria/Babesia 18S rRNA (V4)	Conventional	F - GACACAGGGAGGTAGTGACAAG<br>R - CTAAGAATTTCACCTCTGACAGT	403	[45]
Theileria orientalis MPSP	Conventional	F - CTTTGCCTAGGATACTTCCT<br>R - ACGGCAAGTGGTGAGAACT	776	[49]
Theileria annulata Tams-1	Nested	F1 - GTAACCTTTAAAAACGT<br>R1 - GTTACGAACATGGGTTT<br>F2 - CACCTCAACATACCCC<br>R2 - TGACCCACTTATCGTCC	721<br>453	[50]
Babesia bovis sbp4	Nested	F1 - AGTTGTTGGAGGAGGCTAAT<br>R1 - TCCTTCTCGGCGTCCTTTTC<br>F2 - GAAATCCCTGTTCCAGAG<br>R2 - TCGTTGATAACACTGCAA	907<br>503	[48]
Babesia bigemina RAP1a	Nested	F1 - GAGTCTGCCAAATCCTTAC<br>R1 - TCCTCTACAGCTGCTTCG<br>F2 - AGCTTGCTTTCACAACTCGCC<br>R2 - TTGGTGCTTTGACCGACGACA	879<br>412	[48]
Anaplasma/Ehrlichia 16S rRNA	Conventional	F - GGTACCTACAGAAGAAGTCC<br>R - TAGCACTCATCGTTTACAGC	345	[46]
Anaplasma marginale msp4	Conventional	F - CTGAAGGGGGAGTAATGGG<br>R - GTAATAGCTGCCAGAGATTCC	344	[51]
Ehrlichia ruminantium pCS20	Nested	F1 - ACTAGTAGAAATTGCACAATCYAT<br>R1 - RCTDGCWGCTTTYTGTTCAGCTAK<br>F2 - TGATAACTTGGWGCRRGDARTCCTT<br>R2 - RCTDGCWGCTTTYTGTTCAGCTAK	279	[52]
Coxiella burnetiid 16S rRNA	Conventional	F - ATTGAAGAGTTTGATTCTGG<br>R - CGGCTTCCCGAAGGTTAG	1457	[53]
Rickettsia spp. 16S rRNA	Conventional	F - AACGTCATTATCTTCCTTGC<br>R - AGAGTTTGATCCTGGCTCAG	426	[47]
Rickettsia spp. *gltA*	Conventional	F - GCAAGTATCGGTGAGGATGTAAT<br>R - GCTTCCTTAAAATTCAATAAATCAG-GAT	401	[54]
Rickettsia spp. *ompA*	Conventional	F - GCTTTATTCACCACCTCAAC<br>R - TRATCACCACCGTAAGTAAAT	212	[55]
